# The Extraction of Roots—When Is It Indicated?

**Published:** 1872-03

**Authors:** H. L. Sage


### SELECTED ARTICLES.

### ARTICLE V.

### *The Extraction of Roots—When is it Indicated?*

BY H. L. SAGE.

A widely spread, if not almost universal opinion seems to prevail with dentists—or at least it is usually borne out by practice—that the root of a tooth, if bereft of its crown, might just as well or had better be removed ; that it serves no good purpose, its usefulness being gone; that it is, necessarily, a source of irritation, or that the deleterious effects resulting from its presence in the alveolus more than counterbalances the good derived ; in fact, the idea that the extraction of a root, in any instance, is a loss, in any sense of the word, never occurs to many. And why should it, when they do not hesitate to advise, or at least reluctantly (?) consent, to the extraction of whole colonies of teeth, that might just as well be saved by filling, that they may insert their cheap substitutes? While it is true that many, perhaps a majority, of roots of teeth which are presented to our notice, are so diseased, filthy, and noxious that their presence in the mouth is a positive injury, it is equally true, that they may many times, serve an important purpose, by remaining as sentinels to guard the integrity and prevent the loss of the hard tissues around them, and in other ways be made useful. The maintenance of the alveoli and the organs implanted therein, is sometimes more dependent on the preservation of roots in a healthful condition than is signified by the usual practice with regard to them. But how seldom is any attempt made to retain them by appropriate treatment. That there *are* times when the retention of the roots of teeth, and the prevention of decay therein, is wise and desirable, it is my purpose to show. Hence the inquiry heading this article.

For example: B. consults Dr.———in regard to the feasibility of having an upper partial set of teeth inserted. Most of the teeth back of the cuspidati are yet remaining, while the latter are in good condition. The left central incisor crown is absent, while the root remains. The remaining incisors have cavities in each approximal surface, some of them large, but the majority of medium size. "To make a good thing," he is advised to have all the incisors, including the root, extracted, and a plate with four front teeth inserted. Another, more conservative, would advise the the extraction of the root only.

What would be the *true* course here?

First—Fill the cavities in the incisors thoroughly with gold.

Second—Prepare the root for the reception of a gold crown. But if it is in a doubtful condition—dead—even though it has never made trouble, first ascertain if it is in a state to receive it, which may be done by the insertion of a temporary test-filling in the root of floss silk saturated with creosote, and a crown filling of oxychloride of zinc, or guttapercha. If, after a reasonable time, trouble is experienced, with more or less swelling of the gum, it is obvious, that further treatment must be resorted to before filling with gold.

It may be objected, that a gold crown inserted on a front root would be too conspicuous, disfiguring the individual. Allowing that the objection is a serious one—which I do not, in comparison with the inconvenience and trouble connected with wearing a plate—the root may be prepared for an artificial crown, as detailed in the *Dental Times*, vol. vi. page 36, and thus be protected from decay. But the former method, even, is decidedly preferable to the insertion of a plate of any kind, where natural teeth, or most of them, are remaining; for there is not only some inconvenience attendant upon wearing it, but it has an unfavorable effect upon the remaining teeth, be it ever so nicely adapted to the mouth.

In the act of mastication there is more or less movement of the plate from side to side, which causes a greater or less degree ot irritation of the soft parts of the mouth, producing the loss or absorption of the hard tissues, and thus the teeth lose the support therefrom received, often to such an extent that they elongate, loosen, and drop out. A partial plate, with isolated teeth attached, cannot be worn without, in this indirect way, causing *some* injury to the adjoining natural teeth.

Again, if it produces irritation of the soft parts, the fluids of the mouth become contaminated or changed in character, and thus act upon the structure of the teeth.

In addition to the above cited objections and inconveniences, the difficulty of speaking is increased, and mastication is less perfectly performed than when the natural organs, or parts of organs, are retained, and the shape and contour of the latter restored. The object should be to avoid wearing a plate, if possible.

Again, suppose, in the above cited case, to carry out the illustration, the root is removed. From this cause, also, the integrity of the arch is impaired ; for the loss of the alveolus is, to a certain extent, varying with individual, soon after, or in time, effected by the process of Nature, who upon, its accomplishment, retires from the particular work in that field of operation, seemingly placing a limit on the duty required of the hard tissues in the immediate vicinity of the root, which was to sustain it in position, dead or alive; but this action having ceased upon its extraction, the adjoining teeth have, by its *removal*, also lost, in a measure, their support.

While there is but one objection to a *well* inserted gold crown on the root of a front tooth—its conspicuousness, but which does not hold good in the case of the back teeth—there are many considerations in its favor, besides those which have already appeared.

To the individual concerned, its presence can not be distinguished from that of the adjoining natural crowns, so far as the sense of feeling, comfort, and efficiency in operation is concerned, while of its permanency and durability for years there can be no question, when the operation is thoroughly and *properly* performed. Noticeable instances of the display of good sense now and then appear, in which the individual, in order to save the remnants of the natural teeth, has quarters, thirds, halves, and wholes of lost crowns, replaced with gold, rather than resort to artificial teeth.

The natural functions are, on the whole, more efficiently aided and conserved by the former course than by the latter.

The following. will afford an example of a case in which the extraction of the roots was *not* indicated. In a mouth, with most of the teeth in good condition, attention was directed to the roots of the left inferior six-year molar, the teeth anterior and posterior thereto being sound. The opposing teeth were also sound and serviceable, and would have been entirely so, had not the superior six-year molar been deprived of its antagonizing tooth; so that all that was needed to utilize the entire machinery of that side was to fill the vacancy caused by the loss of the inferior six-year molar.

The roots had formerly been abscessed, treated, and the pulp and crown cavity filled, but all traces of the operation, excepting, perhaps, a few particles of gold in the extreme portion of the root canal, had ceased to be visible.

The decay extended on the buccal and lingual surfaces, about even with the gum, and below it, on the posterior approximal, or distal, a thin anterior proximal wall only being left standing; so that the roots were deprived of very nearly the entire crown, besides being extensively hollowed out by caries.

By the exercise of patience and very much care, the rubber dam was adjusted, though with difficulty, the root prepared for the reception of the gold foundation—one hundred and four grains of gold, light and heavy—and eleven and one-half hours of time employed, and the operation was eminently successful. During the time allotted to the malleting of the gold—an unbroken period of six hours—not a particle of moisture obtruded upon the filling, and the tooth is now as useful as any of its fellows, and is believed to be so secured, so adapted, so condensed, and so finished as to insure its retention for a long time.

There are other cases in which the extraction of roots is not advisable. Persons whose teeth had presented the characteristics of hardness, density, yellowness; of great firmness, with hard, healthy gums, but which are now worn down, by occlusion, nearly to the margin of the latter, should not permit the extraction of the roots, for it is a question whether they are not, on the whole, capable of greater usefulness than a set of artificial dentures would be. We usually find associated with this class of teeth, thick, bulging alveoli and gums, which render the insertion of *serviceable* dentures quite difficult, and the possessor of the former awkard, if not with little patience in the management of substitutes, especially if *he* has passed the prime of life.

But that the extraction of roots *is* indicated when artificial teeth, with the exception of those on pivots, are to be adapted to the mouth, may, with probable truth, be asserted. In most cases, the irritation produced by the insertion of the plate over roots, would be a sufficent reason for their removal, aside from the fact, that most roots are in such a condition as to be offensive and deleterious, Besides, the permanence and usefulness of the substitue, is compromised and hindered by their presence. In most cases, nature is constantly at work for the expulsion of roots; and so it is with *entire* teeth which have lost their antagonists; but the former, if mounted with artificial crowns of gold (when the indications exist for such a course), are as likely to be retained as dead natural antagonizing teeth, which are in a state to be tolerated, for they are then in a condition to perform service, the lack of the ability to do which, had previously placed nature in more formidable antagonism with them, and hence the attempt to expel them from the alveoli.

Again, take a person with teeth, in the main, excellent; who has scrupulous regard to their cleanliness, and is particular in the extreme; with none of the incisors and canines absent; with the crowns of the second superior bicuspids, for instance, gone, but the roots remaining, and not in any manner exerting a deleterious influence in the mouth, and the gums about them healthy.

In such a case, the roots, if sound, may be utilized by building crowns upon them. If this is not done, it is well to fill, at least the pulp cavity, with gold, and thus preserve them, that they may prevent, by their presence, the loss of alveolus.

Many dentists are of the opinion, that incisor and bicuspid crowns of gold can not be rendered permanent; but, having had positive proof to the contrary, I am not disposed to concur with them in this belief. In a case like or similar to the foregoing, the insertion of a plate would seem to be of doubtful expediency, and productive of more injury than benefit.

Even the lateral incisors, which are the smallest teeth in the mouth (if we except the lower central), when decayed to such an extent that the crowns are wanting, may be restored with gold—even the inferior.

If Miss B., makes her appearance with one of the right or left upper or lower lateral incisor roots bereft of its crown, and the other teeth capable of being made useful, it is my duty to advocate such a course, and prepare the root by treatment, if necessary, and the nature of the case will warrant it, for the reception of a gold crown; which, with its *one* objection, of conspicuousness or unnatural appearance, can not overbalance the positive advantages of comfort, natural feeling, ease in speaking, cleanliness, heathfulness, preservation of the hard tissues, the adjoining teeth, etc. This practice may apply, in some cases, not only to one but to several teeth in like condition.

Again, suppose Mr. N., a clergyman, calls. He has an excellent set of natural teeth, in form, structure, firmness, and regular appearance, with a good *physique* to back them. The right superior cuspidatus was filled seventeen years ago, the root included, but three-quarters or more of the crown has, by neglect, or otherwise, been suffered to decay and break down. It looks badly and interferes with speech somewhat, which is unfortunate, especially as he is a public speaker. It is a very firm, healthy (though lifeless) root; at least it has no pulp—or periosteum lining the pulp chamber.

What is the course indicated here?

Everything being in readiness, by previous preparation and confidence in its integrity, commence at the end of the root canal, fill, build down, and restore the shape and contour of the tooth. If well done, there is no question about its permanence. Who would advocate extraction in such a case? To do so would be a grievous blunder, to characterize it by no harsher term.

Those who object to contour fillings must confine their objections to fifths, quarters, and thirds of crowns, in which full restoration to shape and size may not be unqualifiedly essential, and then at the risk of being deemed guilty of the folly of choosing the greatest of two evils—always provided the consent of the patient can be obtained to the exercise of the best judgment of the operator. * * * *
—*Dental Register.*

---

